# Curve Set Feature-Based Robust and Fast Pose Estimation Algorithm

**DOI:** 10.3390/s17081782

**Published:** 2017-08-03

**Authors:** Mingyu Li, Koichi Hashimoto

**Affiliations:** Graduate School of Information Sciences, Tohoku University, Aramaki Aza Aoba 6-6-01, Aoba-Ku, Sendai 980-8579, Japan; koichi@m.tohoku.ac.jp

**Keywords:** bin picking, pose estimation, curve set feature, rotation match feature, pose verification

## Abstract

Bin picking refers to picking the randomly-piled objects from a bin for industrial production purposes, and robotic bin picking is always used in automated assembly lines. In order to achieve a higher productivity, a fast and robust pose estimation algorithm is necessary to recognize and localize the randomly-piled parts. This paper proposes a pose estimation algorithm for bin picking tasks using point cloud data. A novel descriptor Curve Set Feature (CSF) is proposed to describe a point by the surface fluctuation around this point and is also capable of evaluating poses. The Rotation Match Feature (RMF) is proposed to match CSF efficiently. The matching process combines the idea of the matching in 2D space of origin Point Pair Feature (PPF) algorithm with nearest neighbor search. A voxel-based pose verification method is introduced to evaluate the poses and proved to be more than 30-times faster than the kd-tree-based verification method. Our algorithm is evaluated against a large number of synthetic and real scenes and proven to be robust to noise, able to detect metal parts, more accurately and more than 10-times faster than PPF and Oriented, Unique and Repeatable (OUR)-Clustered Viewpoint Feature Histogram (CVFH).

## 1. Introduction

Removing individual objects from an unordered pile of parts in a carrier or box (bin picking) is one of the classical problems of robotics research [1]. Typically, the system consists of a sensor mounted above the box, an industrial robot arm and a processor. As 3D sensors are becoming cost effective, bin picking systems using 3D sensors have been developed in recent years [1,2,3,4]. In this paper, we address the challenge of estimating the poses of parts in bin picking systems with point cloud data efficiently.

Many state-of-the-art pose estimation algorithms incorporate color information with depth information [5,6,7,8]. Hinterstoisser et al. proposed multimodal-LINE (LINEMOD) by combining color images with a dense depth sensor in [7], and Rios-Cabrera et al. [5] proposed Discriminatively Trained Templates (DTT) based on LINEMOD. Hinterstoisser et al. improved LINEMOD in [8] and achieved an average recognition rate of 96.60% and a speed of 119 ms/frame on their ACCV3D dataset. These algorithms can achieve a high recognition rate and speed. However, if color information is not available, the performance declines.

Compared to the daily objects like the ACCV3D dataset of [8], objects in bin picking tasks have two features. One is that the objects usually share the same color. Therefore, the algorithms incorporating color information, such as LINEMOD, cannot present their best performance. Another feature is that many industrial objects are composed of shape primitives such as cylinders, spheres and planes. Some features of the points on these shape primitives are very similar, which makes it more difficult to recognize and localize the industrial objects than daily objects for some algorithms.

One of the promising pose estimation algorithms was proposed by Drost et al. [9]. The algorithm combines an efficient voting scheme with point pair features and does not use color information. Another advantage of the algorithm is that it is robust to occlusion. Due to its pros, many algorithm were proposed based on it. Choi et al. proposed using boundary points with directions and boundary line segments to perform bin picking in order to match planar industrial objects [10]. Birdal et al. incorporated a coarse-to-fine segmentation, a weighted Hough voting, an interpolated recovery of pose parameters and an occlusion-aware ranking method [11] into the original algorithm. Hinterstoisser et al. [12] introduced a better and efficient sampling strategy with modifications to the pre- and post-processing steps and achieved good results on daily objects of the ACCV3D dataset of [8] and the Occlusion Datasetof [13]. Wu et al. [14] also performed bin picking based on [9].

The 3D keypoint descriptors are also capable of pose estimation with only depth information. A set of popular descriptors are available in the Point Cloud Library (PCL) [15]. Rusu et al. introduced the Viewpoint Feature Histogram (VFH) descriptor in [16] and showed better performance than the spin image [17]. Aldoma et al. [18] indicated that VFH was sensitive to noise and occlusions and not capable of estimating a six Degree Of Freedom (DOF) pose. Clustered Viewpoint Feature Histogram (CVFH) was introduced to solve the disadvantages of VFH. A smooth region growing algorithm was applied, and the CVFH descriptor was computed for stable regions. The camera roll histogram was introduced to solve the problem of CVFH invariance to rotations about the camera axis. Aldoma et al. [19] proposed the Oriented, Unique and Repeatable Clustered Viewpoint Feature Histogram (OUR-CVFH) based on CVFH and built local coordinate systems on stable regions to perform 6DOF pose estimation instead of the camera roll histogram. The descriptors can achieve a relatively high speed, but rely heavily on the segmentation result.

There are algorithms recognizing the objects by decomposing point clouds into geometric primitives [3]. Liu et al. [2] developed a multi-flash camera to estimate depth edges. Detected edges are matched with object templates by means of directional chamfer matching. Schnabel et al. [4] detected planes, spheres, cylinders, cones and tori based on RANSAC in the presence of outliers and noise. Holz et al. detected shape and contour primitives to achieve the recognition task [1]. A restriction of the algorithm is that it is only suitable for objects that can be described by contour and shape primitives. It cannot be applied to arbitrary organic objects.

A contribution of this paper is two novel features, the Curve Set Feature (CSF) and the Rotation Match Feature (RMF). The CSF of a point is computed by quantizing the fluctuation of the surface around the point. An RMF is a 360-dimensional feature computed from a CSF for efficient matching. The CSF has the advantage of global features that can describe the surface far from the described point and, at the same time, does not heavily depend on the segmentation result. The CSF is also capable of verifying poses. The matching process is accomplished by nearest neighbor search, therefore being very fast. Another contribution is a fast voxel-based pose verification method to verify a large number of poses and choosing the best poses.

The rest of the paper is organized as follows: Section 2 proposes the curve set feature and the rotation match feature. Section 3 introduces the pipeline of our pose estimation algorithm. Section 4 provides experiments to examine the algorithm, and Section 5 gives the conclusions.

## 2. Curve Set Feature and Rotation Match Feature

In this paper, we denote $s_{i}\in \left\{S\right\}$ for points in the scene cloud, $m_{i}\in \left\{M\right\}$ for points in the model cloud, $n(m_{i})$ for the normal of $m_{i}$, $N_{m}$ for the number of model points, $N_{s}$ for the number of scene points and $N_{select}$ for the number of selected scene points for which we compute the descriptor and match with model points. The model diameter diam(M) is the diameter of the circumcircle of the model. We further denote $SP(s_{i})$ for the visible points within the sphere centered at $s_{i}$ with radius diam(M).

### 2.1. Curve Set Feature

**Figure 1:**
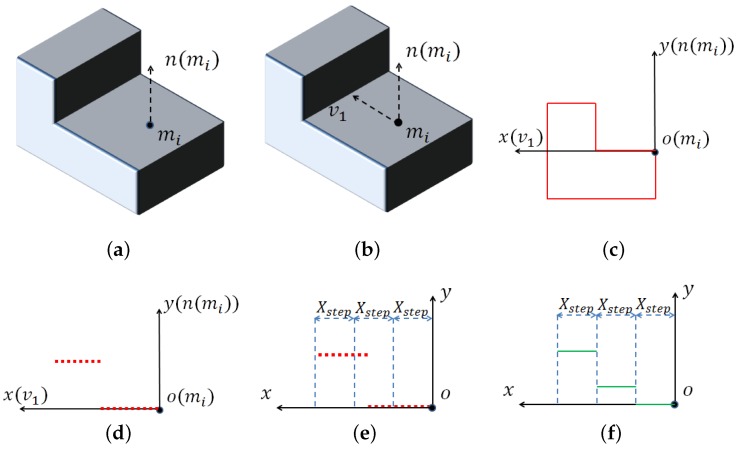
Example of computing a curve feature of point $m_{i}$. (**a**) An L-shaped object with point $m_{i}$ on it and its normal $n(m_{i})$; (**b**) a vector $v_{1}$ starting from $m_{i}$ and perpendicular to $n(m_{i})$; (**c**) build the 2D local coordinate system, and find the points within the plane (red points); (**d**) delete invisible points; (**e**) divide the intervals (**f**) compute the average y values in the intervals.

The element used in our algorithm is a curve feature. A curve feature of a point $m_{i}$ is a quantized 2D curve starting from $m_{i}$ and within the sample plane as the normal $n(m_{i})$, as presented in Figure 1. It is computed by the following steps:

1. Choose a vector $v_{1}$ starting from $m_{i}$ and perpendicular to $n(m_{i})$. Build a 2D local coordinate system whose origin is $m_{i}$; the y axis is $n(m_{i})$, and the x axis is $v_{1}$.
2. All of the points within the local coordinate system whose x value is between zero and diam(M) are denoted as $C_{1}$. Starting from x=0, divide the local coordinate system into small intervals with length $X_{int}$ (in our experiment, we set $X_{int}$ as the integer not smaller than the downsampling size) in the x direction. In every small interval, reserve the point with the largest y value, and delete others from $C_{1}$ to choose visible points.
3. Divide the local coordinate system in the x direction again with a larger length $X_{step}>X_{int}$. For the points of $C_{1}$ within the *n*-th interval, compute the average y value $\bar{y_{n}}$. If there is no point in this interval, set $\bar{y_{n}}=\infty$.
4. The curve feature of point $m_{i}$ in the direction of $v_{1}$ is $f_{1}^{m_{i}}=\left(\bar{y_{1}},\bar{y_{2}},...\bar{y_{D_{1}}}\right)$, $\left(D_{1}=ceil\left(\frac{diam(M)}{X_{step}}\right)\right)$. We further define $f_{1}^{m_{i}}\left[k\right]=\bar{y_{k}}$.

The above steps describe how to compute the curve feature of $m_{i}$ in one direction. To describe the surrounding points of $m_{i}$, we need to compute the curve features in all directions. For a point, we compute a curve feature every one degree. Therefore, every point has $D_{2}=360$ curve features, and the set of these features is the curve set feature of $m_{i}$:

(1) $$F\left(m_{i}\right)=\left(f_{1}^{m_{i}},f_{2}^{m_{i}},...,f_{D_{2}}^{m_{i}}\right)$$

In Step 2, we need to find the points within the plane, but it is time consuming to traverse all points in $SP(m_{i})$ 360 times. Instead, we only traverse $SP(m_{i})$ once before computing the features and assign every point to the plane to which it belongs.

When computing the curve features, we delete invisible points to enable consistency between the scene cloud and the model cloud. In general, the model cloud contains all points of the object, while the scene cloud contains only a part because of occlusion. If we do not consider this difference, the $\bar{y}$ of the model cloud will be smaller than that of the scene cloud. When the object is in different poses, the visible part changes, and we cannot consider so many situations. Therefore, when computing the curve features of a point (both model point and scene point), we assume that the camera’s view direction and the normal of this point are collinear.

### 2.2. Compare Curve Set Features

We define Curve Similarity (CS) to compute the similarity between two curve features:

(2) $$\begin{array}{cc} CS(f_{p}^{m_{i}},f_{q}^{m_{j}}) & =\sum_{k\leq D_{1}}h(f_{p}^{m_{i}}\left[k\right],f_{q}^{m_{j}}\left[k\right],y_{thres})\\ h(x,y,z) & =\left\{\begin{array}{cc} 1 & ∥x-y∥\leq z,x\neq \infty ,y\neq \infty \\ 0 & else \end{array}\right. \end{array}$$

where $y_{thres}$ is the threshold. In our experiment, we set $y_{thres}=0.05\times diam\left(M\right)$.

We further define Curve Set Similarity (CSS) and the Summation of Curve Set Similarity (SCSS) to describe the similarity between two curve set features. Considering the curve features in different directions of a point are different, we need to specify the rotation angle in CSS and SCSS:

(3) $$\begin{array}{c} CSS\left(F\left(m_{i}\right),F\left(s_{j}\right),\alpha \right)=\left(p_{1},p_{2},...p_{D_{2}}\right)\\ p_{k}=CS\left(f_{k}^{m_{i}},f_{k^{\prime }}^{s_{j}}\right)\\ k^{\prime }=\left\{\begin{array}{cc} k+\alpha & k+\alpha \leq D_{2}\\ k+\alpha -D_{2} & else \end{array}\right.\\ SCSS\left(F\left(m_{i}\right),F\left(s_{j}\right),\alpha \right)=\sum_{l\leq D_{2}}p_{l} \end{array}$$

Curve similarity $CS(f_{p}^{m_{i}},f_{q}^{m_{j}})$ is equivalent to matching the local coordinate systems of the curves and counting the number of curve feature elements, which share the same interval index and a similar value. Therefore, if the CS value of two curve features is large, it means these two curves are similar.

**Figure 2:**
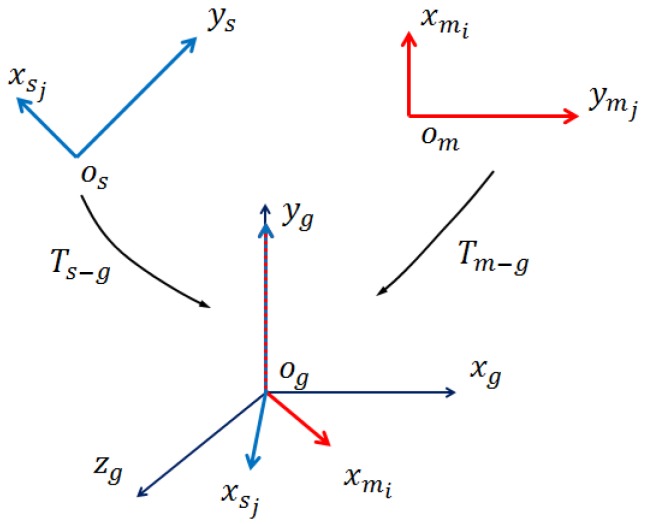
Transformation of the model and scene local coordinate systems [9] proposed. A pose can be computed from Equation (4).

Curve set similarity $CSS(F\left(m_{i}\right),F\left(s_{j}\right),\alpha )$ is equivalent to aligning $m_{i},s_{j}$ and their normals, rotating $F(m_{i})$ around the normal by α and computing the curve similarities with $F(s_{j})$ in every direction, which is shown in Figure 2. The transforming from $F(m_{i})$ to $F(s_{j})$ can be expressed as:

(4) $$\begin{array}{c} s_{j}=T_{s_{j}-g}^{-1}R_{y}\left(\alpha \right)T_{m_{i}-g}m_{i}\\ P\left(m_{i},s_{j},\alpha \right)=T_{s_{j}-g}^{-1}R_{y}\left(\alpha \right)T_{m_{i}-g} \end{array}$$

where $P(m_{i},s_{j},\alpha )$ is the pose, and we borrow this idea from [9]. Therefore, $CSS(F\left(m_{i}\right),F\left(s_{j}\right),\alpha )$ shows the curve similarity in every direction for pose $P(m_{i},s_{j},\alpha )$, and $SCSS(F\left(m_{i}\right),F\left(s_{j}\right),\alpha )$ can be used as a rough pose estimation for $P(m_{i},s_{j},\alpha )$.

The dimension of the curve set features is $D_{1}D_{2}$, which only depends on diam(M) and $X_{step}$. Therefore, the downsampling sizes of the model cloud and the scene cloud can be different as long as the size is smaller than $X_{step}$.

### 2.3. Rotation Match Feature

In pose estimation tasks, the key is to find correspondence between the model and scene. However, it is difficult to search corresponding points using curve set features without any preprocessing. Firstly, the dimension of curve set features is $D_{1}D_{2}$ (usually larger than 1000), which makes it difficult to search efficiently. Secondly, the curve set features are not rotationally symmetric around the normal, and that is why we need to specify α when computing CSS. Therefore, we propose the Rotation Match Feature (RMF) to solve these problems.

The RMF of a point $m_{i}$ is computed by the following steps:

1. Randomly choose a model point $m_{r}$ as the reference point.
2. From α=0, compute $CSS(F\left(m_{i}\right),F\left(m_{r}\right),\alpha )$ every one degree. Then, save the α as $\alpha_{max}\left(m_{i},m_{r}\right)$ when $SCSS(F\left(m_{i}\right),F\left(m_{r}\right),\alpha )$ reaches its maximum value.
3. The RMF of $m_{i}$ is the CSS when $\alpha =\alpha_{max}\left(m_{i},m_{r}\right)$.

(5) $$RMF\left(m_{i},m_{r}\right)=CSS\left(F\left(m_{i}\right),F\left(m_{r}\right),\alpha_{max}\left(m_{i},m_{r}\right)\right)$$

**Figure 3:**
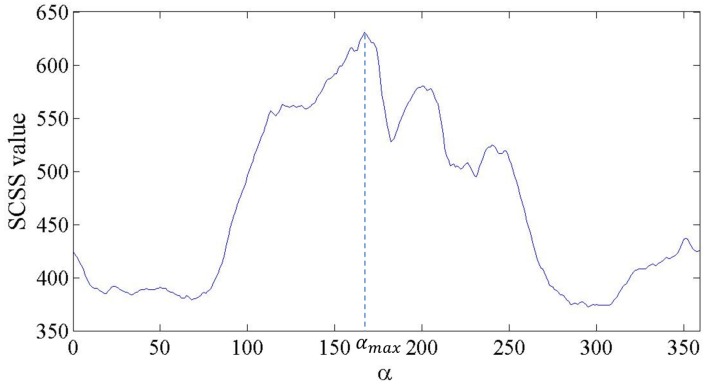
A sample of the Summation of Curve Set Similarity (SCSS) value against α for $\left(m_{i},m_{r}\right)$.

A sample of the SCSS value against α is presented in Figure 3. The aim of selecting model reference points and computing the $\alpha_{max}\left(m_{i},m_{r}\right)$ is to eliminate the rotation degree of freedom and decrease the dimension of the feature. If $m_{i}$ and $s_{j}$ are corresponding points, their RMF features with the same reference point should be close.

Suppose a pair of corresponding points $m_{i}$ and $s_{j}$ are founded, and we need to compute the transformation to match the model to the scene. After aligning the two points and their normals, another rotation $R_{y}\left(\alpha \right)$ around the normal is necessary, and we can decide α by using RMF. When computing RMF of the two points, we match $m_{i}$ and $s_{j}$ to $m_{r}$ based on Equation (4):

(6) $$\begin{array}{cc} m_{r}= & T_{m_{r}-g}^{-1}R_{y}\left(\alpha_{max}\left(m_{i},m_{r}\right)\right)T_{m_{i}-g}m_{i}\\ m_{r}= & T_{m_{r}-g}^{-1}R_{y}\left(\alpha_{max}\left(s_{j},m_{r}\right)\right)T_{s_{j}-g}s_{j} \end{array}$$

Therefore, the transformation from the model to the scene is:

(7) $$s_{j}=T_{s_{j}-g}^{-1}R_{y}\left(\alpha_{max}\left(m_{i},m_{r}\right)-\alpha_{max}\left(s_{j},m_{r}\right)\right)T_{m_{i}-g}m_{i}$$

Instead of only one reference point, we use $N_{r}$ model reference points to improve the recognition rate. Besides, to improve efficiency, we do not traverse all 360 degrees when computing the RMF of scene points. Instead, we compute the SCSS value every five degrees and find the angle $\alpha_{temp\_max}$ with the maximum SCSS value. Then, we check the neighboring angles of $\alpha_{temp\_max}$ and choose $\alpha_{max}$.

## 3. Matching Process

**Figure 4:**
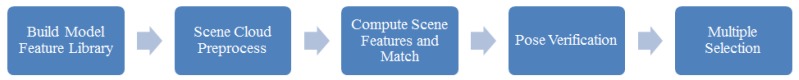
Workflow of the matching process.

The workflow of the matching process is presented in Figure 4.

The matching process consists of five steps: (1) Build the model feature library during the offline stage. Features of the model points are computed and stored in the library for future search and matching, which is introduced in Section 3.2. (2) Scene cloud preprocessing is introduced in Section 3.3. Outliers are removed from the scene cloud, and selected scene points are chosen from the scene cloud. (3) Compute the scene features and match. The features of selected scene points are computed and searched in the model feature library. Model points and scene points sharing similar features compose correspondence pairs, which is shown in Section 3.4. (4) Verify and grade the pairs by pose verification in Section 3.5. The resulting pose is the pair with the highest score. (5) If necessary, multiple poses can be detected in Section 3.6.

### 3.1. Normal Estimation and Modification

Normal estimation is performed by fitting a plane to some neighboring points, and it has been widely used; therefore, we do not introduce it in detail.

**Figure 5:**
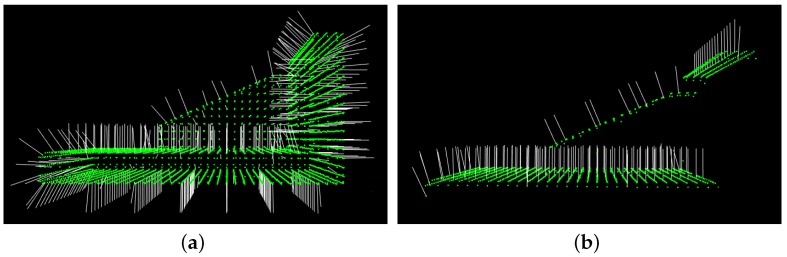
Result of normal sign modification. The green points are cloud points, and white lines are normals. (**a**) Model cloud and its normals; (**b**) scene cloud and its normals; the camera is above the scene cloud.

After estimating the normals, we have to decide the sign of the normals, and in general, there is no mathematical way to solve this problem. For our algorithm, the key is that if the model and scene are correctly matched, the sign of their normals should be consistent. Therefore, we make the normals of the model and the scene cloud point outward from the objects in our algorithm, as presented in Figure 5. Considering that there is always occlusion in the scene cloud, the sign of model normals and of the scene normals is computed in a different way.

A scene cloud is always partially visible, and the viewpoint is always outside the object. Therefore, we define a vector $vs_{i}$ staring from a scene point $s_{i}$ to the viewpoint. The angle between $n(s_{i})$ and $vs_{i}$ should be less than 90${}^{\circ }$. If not, the sign of $n(s_{i})$ changes.

If the model cloud is from a CAD model, the triangle vertices $\left(v_{1},v_{2},v_{3}\right)$ of CAD meshes are always ordered consistently, so that the cross products $\left(v_{1}-v_{2}\right)\times \left(v_{1}-v_{3}\right)$ point either inward or outward. The sign of model normals can be decided by the mesh the pointing inward. If the model cloud is from a 3D sensor, the same method as for the scene cloud can be used.

### 3.2. Build Model Feature Library

**Figure 6:**
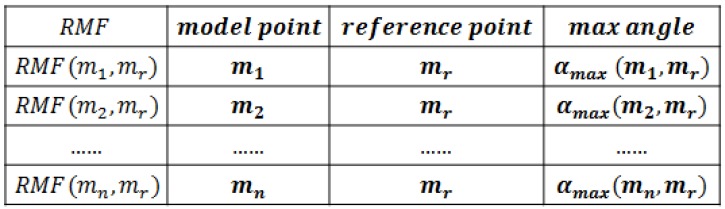
Information stored in the model feature library.

The model feature library is built during offline stage. Suppose we have $N_{m}$ model points and $N_{r}$ reference points, then the library contains $N_{m}N_{r}$ items. Each item contains four pieces of information: model point index, reference point index, RMF and $\alpha_{max}$ of these two points, as shown in Figure 6.

### 3.3. Scene Cloud Preprocess

In real bin-picking tasks, the position of the bin is usually known. Therefore, in order to reduce the computation time and noise points, we remove these points from the scene cloud.

Then, we proceed with a Euclidean segmentation on remaining points. Performance increases by considering only scene points in the same cluster when computing curve features. However, our algorithm does not rely heavily on the segmentation result, and we will show that in Section 4.

Selected scene points are points for which we compute the curve set features and match with model points. The features and the matching process rely on the normals of these points. We found that the normals of the points near boundary points were not reliable. Therefore, a boundary estimation is proceeded on the scene cloud, and scene points that are far away from boundary points are the candidates. Then, we randomly choose $N_{select}$ points from the candidates as selected scene points.

### 3.4. Scene Feature Computation and Nearest Neighbor Search

For a selected scene point $s_{j}$, $RMF(s_{j},m_{r})$ is computed and searched in the model feature library using the Fast Library for Approximate Nearest Neighbors (FLANN) [20].

Suppose we search $RMF(s_{j},m_{r})$ in the library and get $RMF(m_{i},m_{r})$. $s_{j}$ and $m_{i}$ may be corresponding points because they share a similar RMF. We match $m_{i}$ to $s_{j}$ as described in Section 2.3 and Equation (7).

Following [9], here, we call the transformation pair $\left(m_{i},s_{s},\alpha \right)$ a local coordinate. Because of noise, occlusion and other factors, the model point with the nearest RMF may not be the corresponding point of the scene point. Therefore, we search Knn nearest model RMF for every scene RMF. The transformations (poses) between the model points and scene points are saved for pose verification. There are in total $N_{select}N_{r}Knn$ poses to verify. The process is presented in Algorithm 1.

 **Algorithm 1:** Compute scene feature and match 
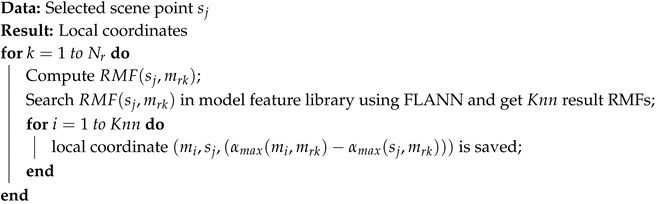


### 3.5. Pose Verification

The number of poses (local coordinates) to verify from the last stage is large. As mentioned before, the SCSS value can be treated as a rough pose verification method, and we can use it to improve the verification efficiency. Given a local coordinate $\left(m_{i},s_{j},\alpha \right)$, we compute $SCSS(F\left(m_{i}\right),F\left(s_{j}\right),\alpha )$ to evaluate the performance. After computing the SCSS value for all of the local coordinates, we select top $N_{p}$ from them for the next verification.

An idea of pose verification is to transform the model cloud into the scene space. Then for every model point, the nearest scene point is searched and the distance between these two points and the angle between their normals are computed. If the distance and the angle are smaller than the specified threshold, this model point is considered to be fitted. If the number of fitted points is sufficiently large, the pose is considered to be correct [21]. This method is intuitive and effective, but time consuming, because it needs to search the nearest scene point for every model point and every pose.

**Figure 7:**
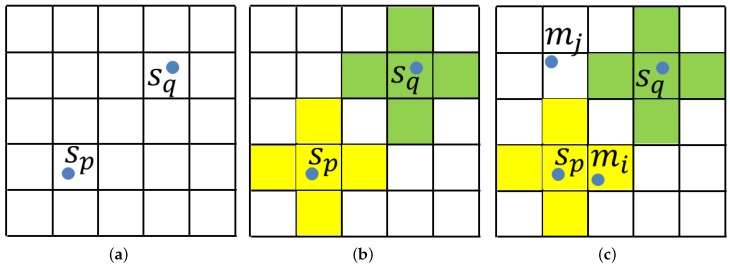
An example of searching nearest scene point. (**a**) Scene points $s_{p}$, $s_{q}$ and the voxels. (**b**) The values of seed voxels and surrounding voxels change to the scene point index. The values of yellow voxels are *p*, and those of green voxels are *q*. The values of white voxels are −1. (**c**) Two transformed model points $m_{i}$, $m_{j}$. The value of the voxel $m_{j}$ in is −1; therefore, there is no valid nearest scene point of $m_{j}$. The value of the voxel $m_{i}$ in is *p*; therefore, the nearest scene point of $m_{i}$ is $s_{p}$.

**Figure 8:**
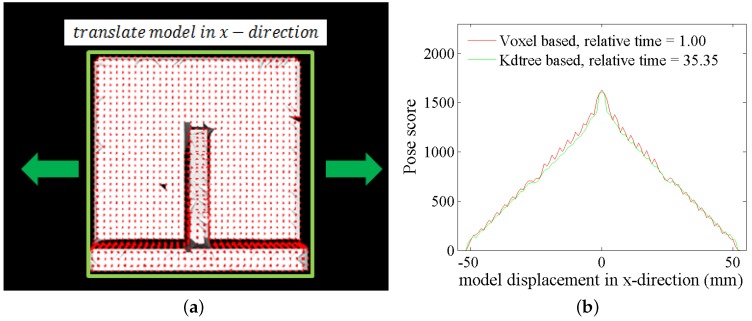
(**a**) The gray part is the scene cloud with the triangle mesh, and the red points are the model cloud. The model is translated in the x direction. (**b**) The pose score against displacement of the model cloud by our verification method and the kd-tree-based method. The score difference between the two methods is small, and our method is more than 30-times faster.

The key in pose verification is to search the nearest scene point for transformed model points efficiently. To achieve this, we divide the scene space into small cubic voxels with length $L_{voxel}$. The edges of the voxels are parallel to the axes of the scene coordinate system. At first, the values of all voxels are −1. The 3D coordinate of a voxel center is converted to three non-negative integers by Equation (8):

(8) $$\begin{array}{c} x_{int}=INT\left(\frac{x_{voxel}-min\_x}{L_{voxel}}\right)\\ y_{int}=INT\left(\frac{y_{voxel}-min\_y}{L_{voxel}}\right)\\ z_{int}=INT\left(\frac{z_{voxel}-min\_z}{L_{voxel}}\right) \end{array}$$

where $\left(x_{voxel},y_{voxel},z_{voxel}\right)$ is the coordinate of the voxel center, min_x,min_y,min_z are the minimum coordinate components of the scene cloud and $x_{int},y_{int},z_{int}$ are the integers. The voxel is accessed through these three integers. Then, for every scene point $s_{j}$, Equation (8) is used to find the voxel that $s_{j}$ is in, and this voxel is a seed voxel $Sv_{j}$. The values of $Sv_{j}$ and surrounding voxels within the sphere centered at $Sv_{j}$ with radius $Radius_{seed}$ change to the index of the scene point *j*. The constant $Radius_{seed}$ is set as the distance threshold (in our experiment, $Radius_{seed}=2$ mm). In the verification, for a transformed model point $m_{i}$, we access the voxel $m_{i}$ in using the same method, and the value of the voxel is the index of the nearest scene point. If the value is −1, it means the distance from the nearest scene point is larger than the threshold, and further verification is unnecessary, as presented in Figure 7. If the transformed model point $m_{i}$ finds a valid nearest scene point $s_{p}$ and the angle between their normals is less than a threshold, $m_{i}$ is a fitted model point. The score of a pose is the fitted model point number. By using this method, we can verify poses efficiently, as presented in Figure 8. The pose score computed from our method and kd-tree is very similar, but our method is more than 30-times faster than kd-tree. After verifying all of the poses, the final pose is the one with the highest score. The verification process is presented in Algorithm 2.

 **Algorithm 2:** Pose verification 
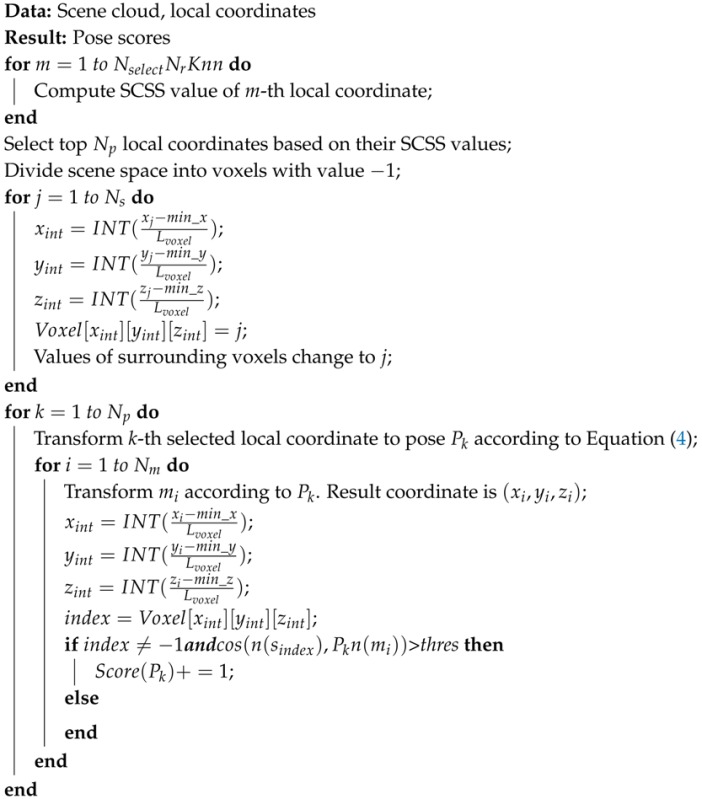



### 3.6. Multiple Pose Detection

Sometimes, it is necessary to detect multiple poses in one scene, and our algorithm is capable of that. During pose verification, a large number of poses was evaluated. These verification results are used by the following steps:

1. Rank all of the poses with their scores.
2. Suppose $P_{1}$ is the first selected pose. Transform the model cloud into scene space according to $P_{1}$.
3. For every transformed model point, check whether the value of the voxel it is in is −1. If not, change the value of all of the voxels sharing the same value with this voxel to −1.
4. Verify the poses with a high grade in Step 1, and choose the pose $P_{2}$ with the highest grade. $P_{2}$ is the new pose.

This is actually to delete the scene points corresponding to the old pose and to select the new pose. If we do not delete the points, we will always get the same pose.

## 4. Experiment

**Figure 9:**
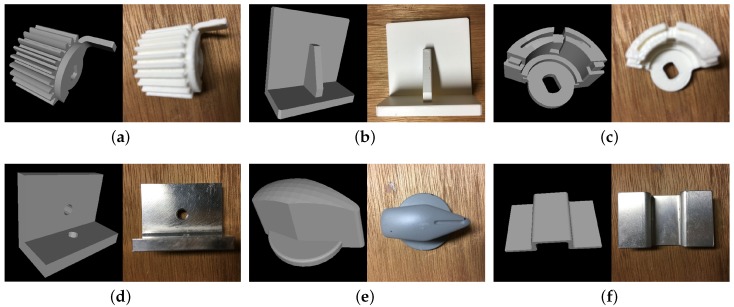
Models used in the experiment. (**a**) Gear, diam(M)=79 mm; (**b**) L-shaped part, diam(M)=73 mm; (**c**) magnet, diam(M)=59 mm; (**d**) metal L part, diam(M)=53 mm; (**e**) switch, diam(M)=49 mm; (**f**) metal bulge, diam(M)=57 mm.

We evaluated our algorithm against a large number of synthetic and real scenes. Six industrial parts were used in our experiment. The models and their diam(M) are shown in Figure 9. We were interested in the recognition rate and speed of the algorithm. Every resulting pose was considered to be correct if the error was less than the specified threshold. In our experiment, the threshold was set to diam(M)/10 for the translation and 5${}^{\circ }$ for the rotation.

For all experiments, We set $X_{step}$ as 3 mm, and the downsampling size of the model cloud and the scene cloud was smaller than $X_{step}$. The default values of the selected scene point number and reference model point number were $N_{select}=50$ and $N_{r}=20$. We compared our algorithm with Drost PPF [9], denoted as **PPF**, and OUR-CVFH [19], denoted as **OUR-CVFH**. The resulting poses of all three algorithms were refined by ICP. All given timings contain the whole process including the normal estimation, boundary estimation, matching and ICP refinement. The algorithms were implemented in C++ and run on an Intel Core i7-4810MQ CPU with 2.8 GHz and 8 GB RAM.

### 4.1. Synthetic Scenes

**Figure 10:**
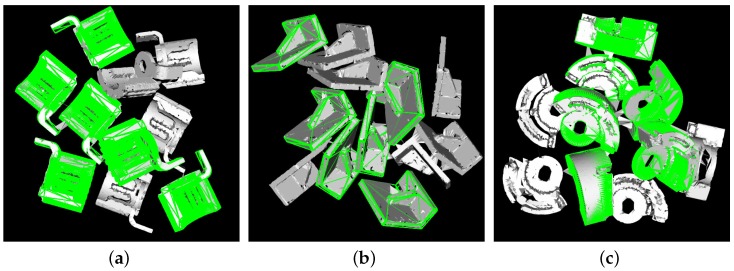
Detection results of synthetic scenes of (**a**) Gear (**b**) L-shaped part (**c**) Magnet. The gray part is the scene cloud with the triangle mesh, and the green frameworks show the poses. All of the resulting poses shown are correct.

We firstly evaluated our algorithm against synthetic scenes. The scenes were generated with multiples of the same object in every scene using the simulator in [22]. One hundred synthetic scenes were generated for every model, and the number of objects in every scene varied from 7–12. Then, occluded points were removed based on the viewpoint. We ran our algorithm four times. The default parameters were used in the first set of experiments, and a smaller $N_{select}=20$ and $N_{r}=5$ were used in the second set. In order to measure the influence of segmentation, we ran the algorithm without Euclidean segmentation using default parameters and fast parameters in the third and fourth set, respectively. The four experiment results are denoted as **CSF Default**, **CSF Fast**, **CSF No Seg Default** and **CSF No Seg Fast** respectively. The recognition rate and speed of the algorithms on every model are presented in Table 1 and Table 2, respectively. Some experiment results are presented in Figure 10. It is seen that when using default parameters, our algorithm achieved a recognition rate of 97.36%. When performed at high speed, our algorithm still gave a higher recognition rate than OUR-CVFH and PPF, and at the same time, it was more than 10-times faster than OUR-CVFH and 35-times faster than PPF.

When using default parameters without Euclidean segmentation, the recognition rate and speed do not change greatly. For fast parameters, the segmentation can improve the recognition rate of the algorithm. As stated in Section 3.3, our algorithm does not depend heavily on segmentation. Using fast parameters without segmentation, our algorithm still performed with a recognition rate of 82.69%.

**Figure 11:**
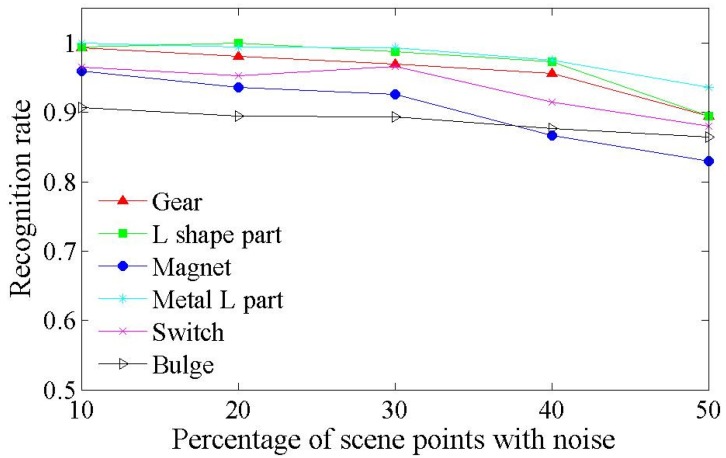
Recognition rates against the percentage of scene points with noise of the six models. Our algorithm still performs with a high recognition rate when severe noise is applied.

**Figure 12:**
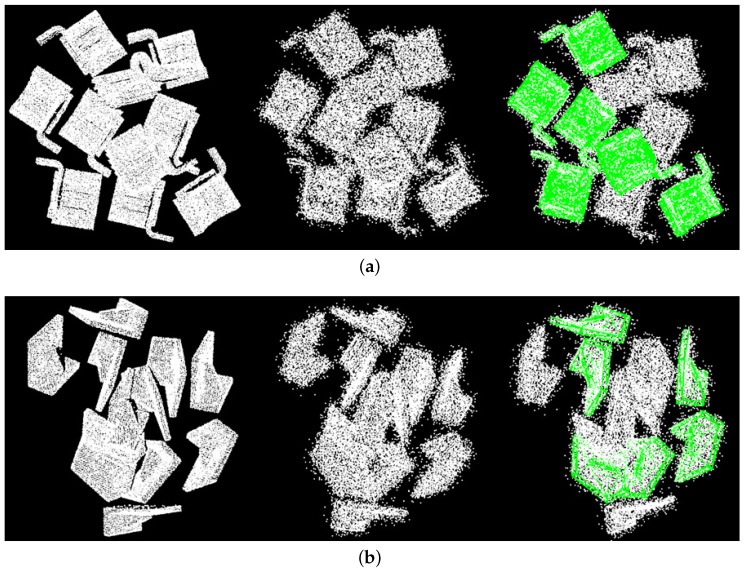
Detection of clouds with noise of (**a**) Gear (**b**) L-shaped part. Left: origin cloud; middle: cloud with noise; right: detection results. All of the resulting poses shown are correct.

We then tested our algorithm against noise. Gaussian noise with a standard deviation of σ=0.05diam(M) was applied on a part of the points (10–50%) in the synthetic scenes. Then, our algorithm was applied on the scenes using default parameters with different percentages of noise points. The performance is presented in Figure 11 and some detection results are presented in Figure 12.

It is seen that our algorithm worked well against noise. When the noise was added on 50% of the scene points, the worst recognition rate was 83.00% for magnet, and an overall recognition rate of over 88.36% was achieved.

### 4.2. Real Scenes

**Figure 13:**
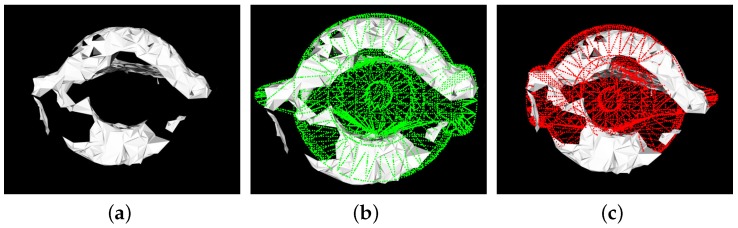
(**a**) The cloud of the switch in the real scene; (**b**) a possible pose; (**c**) another possible pose; Because of the occlusion and noise in the real scenes, it is very difficult to distinguish which pose is correct and to make ground truth poses. Therefore, we did not experiment on switch.

We tested our algorithm for real 3D data scanned with our 3D sensor. We did not experiment on switch because in real scenes, sometimes, the part had two possible poses, and it was difficult for us to distinguish which was correct, as presented in Figure 13.

**Figure 14:**
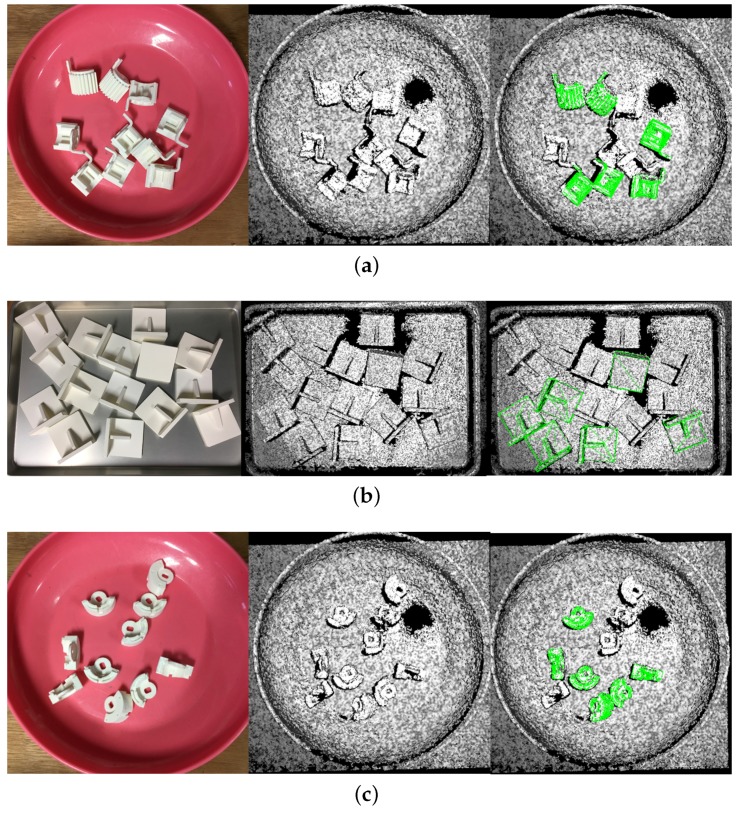
Detection results of the real scenes of (**a**) Gear (**b**) L-shaped part (**c**) Magnet. Left: pictures of the real scenes; middle: clouds of the scenes; right: detection results. All poses shown are correct.

Firstly, we performed quantitative evaluation on the gear, L-shaped part and magnet. We took 25 scenes for each part, and the ground truth poses of the objects were made manually. The same as the simulation experiment, six poses were detected in every scene. The performance of the algorithms were presented in Table 3 and Table 4, respectively, and some results are presented in Figure 14.

**Figure 15:**
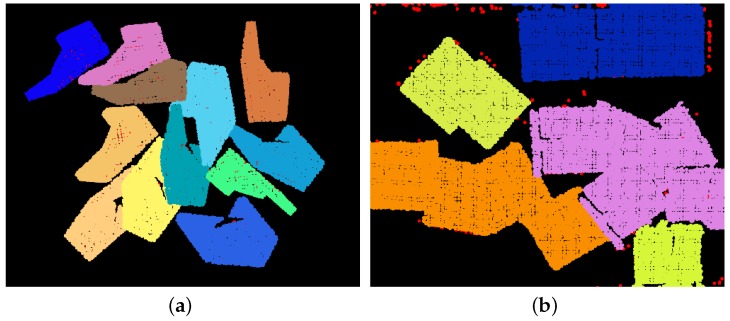
Euclidean segmentation result of the cloud. Points of different clusters have different colors. The segmentation result of the real scene is worse than that of the synthetic scene. (**a**) Result of a synthetic scene; (**b**) result of a real scene.

Though OUR-CVFH presented good results in the synthetic experiment, it performed with the worst recognition rate on the L-shaped part. This is mainly because the segmentation in real scenes was not good enough. OUR-CVFH performs Euclidean segmentation before recognition, and as shown in Figure 15, the segmentation result of the real scene was worse than that of the simulation scene. For gear and magnet, OUR-CVFH performed better because the clouds were easier to segment. Compared to OUR-CVFH, our algorithm is more robust to noise and the failure of segmentation.

**Figure 16:**
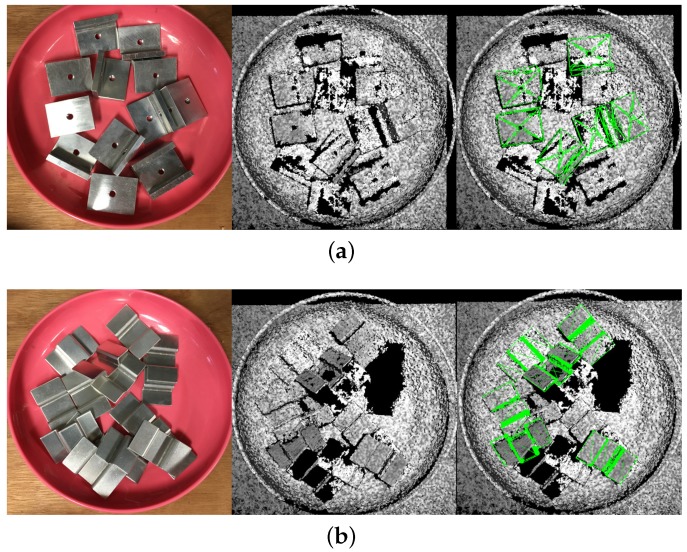
Detection result on metal parts (**a**) Metal L part (**b**) Bulge. Left: pictures of the real scenes; middle: clouds of the scenes; right: detection results. All poses shown seem correct.

The metal L part and bulge are metal parts, and we performed qualitative evaluation on these two parts. Some results are presented in Figure 16. It is seen that our algorithm can estimate metal parts when the lost point number is not very large.

## 5. Conclusions

This study proposes a 6D pose estimation algorithm for a robotic bin picking system. Two features, CSF and RMF, are proposed to describe and match scene points with model points. To improve the efficiency of the pose verification method, we divide the scene space into voxels to replace the kd-tree. Our algorithm was evaluated against a large number of synthetic and real scenes and a high recognition rate and efficiency were presented. Our algorithm is also proven to be robust to noise, heavily cluttered scenes and able to detect metal parts. However, the performance of our method tends to decline for occluded objects because the occlusion causes a change of RMF.

## Figures and Tables

**Table 1:** Recognition rate of the algorithms on synthetic scenes.

Models	CSF Default	CSF Fast	CSF No Seg Default	CSF No Seg Fast	OUR-CVFH [19]	PPF [9]
Gear	**97.67%**	91.67%	96.17%	82.17%	**97.67%**	43.33%
L-shaped part	**100.00%**	99.83%	98.00%	56.33%	94.50%	79.83%
Magnet	**96.00%**	93.17%	95.50%	84.17%	73.33%	87.83%
Metal L part	**99.83%**	88.50%	**99.83%**	88.50%	82.50%	97.33%
Switch	95.33%	91.00%	**97.83%**	90.83%	65.50%	96.33%
Bulge	**95.33%**	93.33%	94.67%	94.17%	89.33%	38.83%
Average	**97.36%**	92.92%	97.00%	82.69%	83.84%	73.92%

**Table 2:** Speed of the algorithms on synthetic scenes (millisecond/object).

Models	CSF Default	CSF Fast	CSF No Seg Default	CSF No Seg Fast	OUR-CVFH [19]	PPF [9]
Gear	215	78	225	80	1327	2579
L-shaped part	167	60	199	77	1078	2249
Magnet	260	91	233	98	1012	4266
Metal L part	167	70	199	73	553	1525
Switch	245	65	219	107	750	4297
Bulge	185	62	192	69	445	979
Average	207	71	211	84	861	2649
Relative time	2.92	1.00	2.97	1.18	12.12	37.31

**Table 3:** Recognition rate of the algorithms on real scenes.

Models	CSF Default	CSF Fast	CSF No Seg Default	CSF No Seg Fast	OUR-CVFH [19]	PPF [9]
Gear	**87.33%**	78.00%	**87.33%**	71.33%	75.33%	74.67%
L shape part	**96.00%**	84.00%	94.67%	75.33%	27.33%	60.00%
Magnet	90.67%	78.00%	**95.33%**	72.67%	62.67%	86.67%
Average	91.33%	80.00%	**92.44%**	73.11%	55.11%	73.78%

**Table 4:** Speed of the algorithms on real scenes.

Models	CSF Default	CSF Fast	CSF No Seg Default	CSF No Seg Fast	OUR-CVFH [19]	PPF [9]
Gear	299	214	287	213	2010	1669
L shape part	196	103	201	114	1845	2811
Magnet	297	180	286	193	1995	2429
Average	264	166	258	173	1950	2303
Relative time	1.59	1.00	1.55	1.04	12.26	14.48
